# Drinking patterns and antidepressant medication – a prospective register-linked study among 40 to 60-year-old employees

**DOI:** 10.1007/s00127-025-02996-z

**Published:** 2025-09-27

**Authors:** Aino Salonsalmi, Jouni Lahti, Eero Lahelma, Ossi Rahkonen, Tea Lallukka

**Affiliations:** 1 Department of Public Health, University of Helsinki, PO Box 20 (Tukholmankatu 8 B), 00014 Helsinki, Finland; 2 Finnish Institute for Health and Welfare, Helsinki, Finland

**Keywords:** Alcohol drinking, Drinking behavior, Binge drinking, Depression, Antidepressant medication

## Abstract

**Purpose:**

To examine the association between drinking patterns and subsequent antidepressant medication using register-linked data.

**Methods:**

The Helsinki Health Study survey (2000–02) of 40- to 60-year-old employees was linked with antidepressant medication data from registers of the Social Insurance Institution of Finland. Drinking patterns included weekly amount of drinking, binge drinking and problem drinking. Cox regression analysis was used to calculate hazard ratios (HR) for the first antidepressant medication purchase during five-year follow-up. Gender, age, occupational position, marital status, relative weight, smoking and leisure-time physical activity were included as covariates. The study included 5727 employees with no antidepressant purchase during 3 years preceding the baseline.

**Results:**

Heavy drinking was associated with an increased risk of antidepressant medication compared to moderate drinking (HR 1.53, 95% CI 1.18–1.99). Non-drinkers (1.43, 1.03–1.97), occasional binge drinkers (1.27, 1.06–1.52) and frequent binge drinkers (1.35 (1.02–1.77) showed an association with antidepressant medication compared to non-bingeing drinkers. Problem drinking was associated with antidepressant medication (1.84, 1.54–2.19). The associations remained after adjusting for occupational position and for marital status. The associations concerning heavy drinking and problem drinking remained also after adjusting for relative weight and health behaviours. An additional analysis among participants with prior antidepressant medication before baseline, showed no associations between drinking patterns and subsequent antidepressant medication during the follow-up.

**Conclusion:**

Alcohol drinking is associated with antidepressant medication among employees. The association is not limited to problem drinking. Paying attention to heavy, binge and problem drinking might help prevent depression.

**Supplementary Information:**

The online version contains supplementary material available at https://doi.org/10.1007/s00127-025-02996-z.

## Introduction

Depression is a major burden to health and well-being worldwide [1]. A meta-analysis covering the years from 1994 to 2014 estimated that the lifetime prevalence of depression was 10.8% [2]. The prevalence of depression was higher when measured by self-report instruments than clinical assessments [2]. Depression is typically treated with antidepressant medication [3] and in Finland the use of antidepressant medication has increased in the 2000 s [4]. Although antidepressant medication is prescribed for other conditions than depression, such as migraine and chronic pain in addition to depression, depression is the most common indication followed by other psychiatric indications often overlapping with depression [5].

Previous evidence suggests a positive association between alcohol drinking and depression [6–8]. Early evidence of the association was mostly based on patients with alcohol problems in psychiatric inpatient settings largely excluding individuals with less severe alcohol problems and general populations [6]. Subsequent studies on general populations have shown inconclusive results. Diverse measures of alcohol drinking and depression might contribute to the inconclusive evidence. A recent meta-analysis analyzing 42 longitudinal studies reported that alcohol use disorder was associated with an increased risk of depressive symptoms [8]. Regarding alcohol intake the results were more uncertain. Heavy drinking was associated with depressive symptoms but only when compared to non-heavy drinkers whereas no significant association was observed when compared to non-drinkers [8]. Considering the possibility of publication bias and confounding factors the association became non-significant [8]. Many disease outcomes are impacted causally by alcohol, often in dose-response fashion [9]. In addition, previous studies emphasize the importance of heavy drinking episodes to alcohol-related diseases [9]. Studies on the associations between binge drinking and depression are, however, scarce. A previous Finnish study found that binge drinking was associated with depression five years later [10].

Most studies examining general populations have measured depression with various self-report instruments such as the depression subscale of the General Health Questionnaire [11], Beck Depression Inventory [10], the Center for Epidemiologic Studies Depression Scale [12] or by structural interviews [13]. Studies using register-based outcomes such as antidepressant medication and hospital discharge register have been scarce. Compared to self-report instruments and structural interviews, which might overestimate the prevalence of depression [2] they are likely to capture severe forms of depression and portray physician’s assessment. However, a Danish study found that drinking over six units per day was not associated with antidepressant medication prescription compared to drinking 0.1-1 units per day when adjusting for age, gender, smoking status, cigarettes per day, physical activity, education, income, civil status, body mass index and plasma C-reactive protein [14]. No association was found either for hospitalization or death with depression [14]. Two Spanish studies using an outcome combining self-reported depression and self-reported use of antidepressant medication reported an increased risk of depression among non-drinkers [15, 16]. A Finnish study included information from hospital discharge register and found that both heavy drinking and binge drinking were associated with hospitalization due to depression [10].

Previous evidence suggests that associations between alcohol drinking and depression are stronger among women than men [8, 17] and a previous study showed a stronger risk among women when focusing on major depression, but when including less severe cases associations were found also among men [18]. Individuals in low socioeconomic positions might have a greater risk of depressive symptoms when consuming high amount of alcohol compared to individuals in higher positions [19]. In addition, marital status [20, 21], relative weight [22, 23] and health behaviours such as smoking and physical inactivity [24, 25] have been associated with both drinking patterns and depression and might thus contribute to the associations. Alcohol drinking and depression often co-exist and potentiate one another, and their association appears bidirectional with alcohol drinking increasing the risk of depression and vice versa [9]. People might try to relieve the symptoms of depression with alcohol drinking which might in turn deteriorate mood disorders and become medicated by antidepressants in the long run. In addition, same hereditary and environmental risk factors might predispose to both alcohol drinking and depression. Thus, it is important to consider information of previous depression when examining the associations.

This study aimed to examine whether alcohol drinking was associated with subsequent antidepressant medication among employees. Drinking patterns were examined with measures covering the weekly amount of drinking, binge drinking and problem drinking. Age, gender, occupational position, marital status, relative weight, smoking and physical inactivity were included as covariates. Employees with antidepressant medication within three years preceding the baseline were excluded from the main analyses and examined separately in an additional analysis.

### Data and methods

This study is part of the Helsinki Health Study on employees of the City of Helsinki [26]. The City of Helsinki with its 37 000 employees is the largest employer in Finland [27]. The majority of the employees are women as is the case elsewhere in the Finnish municipal sector. The jobs include diverse manual and non-manual jobs such as nurses, bus drivers, garden workers, child minders, lawyers and doctors.

The data on drinking patterns and covariates were derived from the questionnaires mailed in 2000, 2001 and 2002 to employees who turned 40, 45, 50, 55 or 60 during those years. Altogether 8960 (78% women) out of 13 346 employees participated and the response rate was 67%. Data on purchases of prescribed reimbursed antidepressant medication were derived from the Social Insurance Institution of Finland among participants who gave written informed consent to the data linkage (74%). Participants with antidepressant medication during 3 years preceding the baseline (*n* = 732) were removed from the main analysis and an additional analysis was performed among them. Antidepressant purchases were followed until the first purchase, death (*n* = 48) or until the end of follow-up time of five years, which ever came first. The mean follow-up time was 4.7 years. Dates of deaths were retrieved from Statistics Finland (the Causes of Death Register). The linked data consists of 5852 participants. After exclusions due to being pregnant at baseline (*n* = 15) and missing information on covariates the analytical sample included 5727 employees.

The Ethics Committee of the Department of Public Health at the University of Helsinki and the health authorities at the City of Helsinki approved the study and the Social Insurance Institution of Finland and Statistics Finland gave permission to the register linkages.

### Drinking patterns

There were three questions with seven response alternatives concerning average use of different kinds of alcoholic beverages namely the use of beer or cider, wine or other mild beverages and spirits. From these data weekly average approximate volume of alcohol use was calculated defining alcohol unit as 12 g of pure alcohol. The cut-point for heavy drinking was set to 12 or more units per week for women and for 23 or more units per week for men according to the Finnish guidelines of heavy alcohol drinking [28].

Binge drinking was inquired with a question asking how often the respondent drank six or more alcohol units at a time. The respondent could choose from six alternatives the best fitting option. Those opting ‘never’ were classified as non-binge drinkers, those opting ‘less often than once per month’ or ‘once per month’ formed the second group and those opting ‘once per week’, a couple of times per week’ or ‘daily or almost daily’ formed the third group. In addition, non-drinkers were separated from non-binge drinkers according to another question inquiring the frequency of drinking. The categories were thus ‘non-drinkers’, ‘non-bingeing drinkers’, ‘binge drinking once per month or less often’ and ‘binge drinking once per week or more often’.

Life-time problem drinking was measured by the CAGE-scale [29], which consists of four questions: Have you ever thought about cutting down your drinking? Have you ever been annoyed by criticism about your drinking? Have you ever felt guilty because of your drinking? Have you ever needed an eye-opener? Each positive answer adds one point to a summary score ranging from zero to four. Cut-point for problem drinking was set to two points for women and to three points for men.

### Antidepressant medication

The Finnish Prescription Register maintained by the Social Insurance Institution includes records of all prescribed medication purchases reimbursed to Finnish residents in non-institutional settings. Medication purchases were classified according to the Anatomical Therapeutic Chemical (ATC) system by WHO [30]. Antidepressant medication consisted of the group N06A. The quantity of purchased medication as defined daily doses (DDD) was used for descriptive purposes. The outcome measure of the study was the first purchase of prescribed, reimbursed antidepressant medication. In all 12% (673) of the participants without prior antidepressant medication purchase had at least one record of prescribed reimbursed medication purchases during the five-year follow-up.

### Covariates

Data on covariates came from the baseline survey with the exception for occupational position which was derived from the employer’s register among those participants who consented for the data linkage. Age was included as a covariate. Occupational position was divided into four groups: ‘manual workers’, ‘routine non-manual workers’, ‘semi-professionals’ and ‘managers and professionals’. Marital status was divided into three categories: ‘single’, ‘married or cohabiting’ and ‘divorced or widowed’. Relative weight was measured by body mass index (BMI) calculated from self-reported data of weight and height (weight/height^2^). BMI was divided into three groups: under 25, between 25 and 30 and above 30 kg/m^2^. Leisure-time physical activity was measured by four questions from which metabolic equivalent tasks (MET) were calculated. Employees having fewer than 14 MET hours per week were classified as being inactive and others as active [31]. Smoking was classified into current smoking and non-smoking.

### Statistical methods

Mean DDDs and their standard deviations were calculated for descriptive purposes. The associations between drinking patterns and antidepressant medication were examined by Cox regression analysis with first purchase of antidepressant medication as outcome. Moderate drinkers, not bingeing drinkers and non-problem drinkers served as reference categories since previous studies suggest that non-drinkers have an elevated risk of depression compared to moderate drinkers and non-bingeing drinkers [11, 14–16]. Interactions for gender in the models were tested and since there were no significant interactions women and men were combined in the analyses. First, we fitted base models adjusted for age and gender. Then occupational position, marital status, and finally relative weight and health behaviours were one by one in turns added to the model already including age and gender. The results are presented as hazard ratios (HR) and their 95% confidence intervals. The proportional hazards assumption was examined using Schoenfeld residuals [32] and confirmed.

The associations between drinking patterns and antidepressant medication were also analysed among participants with prior antidepressant medication using the same reference categories. In these analyses the proportional hazards assumption was violated and thus logistic regression analysis was performed instead. The results are presented as odds ratios and their 95% confidence intervals.

The SAS statistical program version 9.4 was used in performing the analyses (SAS Institute Inc., Cary, NC, USA).

## Results

**Table 1:** The distributions of drinking patterns (n, %). The prevalence of antidepressant medication and mean defined daily doses (DDDs) by drinking patterns

	*n*	%	% with antidepressant medication	Mean DDD (s.d.)
*Weekly amount of drinking*				
0	359	6	13	58 (282)
0.5–11.5 (women)/22.5 (men)	4969	87	11	41 (193)
12 (women)/23 (men) or more	380	7	17	68 (256)
*Binge drinking*				
Non-drinker	325	6	14	58 (286)
No bingeing	2167	39	10	37 (187)
Less often than once a week	2522	45	13	46 (201)
Once a week or more often	609	11	12	50 (236)
*Problem drinking*				
No	4627	83	10	38 (189)
Yes	940	17	18	73 (269)

s.d. standard deviation

### Study population

The vast majority of the participants consumed alcohol. (Table 1) Of the participants 7% were heavy drinkers measured by weekly amount of drinking. Binge drinking and lifetime problem drinking were common: Of the participants 56% were binge drinkers. Of the participants 11% were binge drinking at least once per week and 17% were problem drinkers. The prevalence of antidepressant medication varied by drinking patterns, with non-drinkers, heavy, binge and problem drinkers having the highest prevalence as well as higher DDDs.

**Table 2:** The distributions of covariates (n, %) and prevalence (%) of antidepressant medication use

	*n*	%	% with antidepressant medication
*Gender*			
Woman	4446	78	12
Men	1281	22	10
p			0.0005
*Age*			
40	1167	20	14
45	1217	21	11
50	1228	21	14
55	1402	24	11
60	713	12	8
p			< 0.0001
*Occupational position*			
Manual workers	809	14	9
Routine non-manual workers	1945	34	14
Semi-professionals	1121	20	10
Managers and professionals	1852	32	11
p			0.0003
*Marital status*			
Single	692	12	13
Married or cohabiting	4127	72	11
Divorced or widowed	908	16	15
p			0.0003
*BMI*			
Under 25	2953	52	11
25–30	1966	34	12
Above 30	808	14	13
p			0.1227
*Physical activity*			
Active	4384	77	11
Inactive	1343	23	15
p			< 0.0001
*Smoking*			
No	4448	78	11
Yes	1279	22	15
p			< 0.0001

The distributions of covariates and prevalence of antidepressant medication by covariates are presented in Table 2. Antidepressant medication was more common among women, among routine non-manual workers, among single and divorced or widowed employees, among physically inactive employees and among smokers.

**Table 3:** The associations between drinking patterns and subsequent antidepressant medication. Hazard ratios and their 95% confidence intervals

	Model 1 = Age + gender	Model 1 + occupational position	Model 1 + marital status	Model 1 + bmi, smoking and physical activity
*Weekly amount of drinking*				
0	1.20 (0.89–1.62)	1.21 (0.89–1.63)	1.20 (0.89–1.62)	1.23 (0.91–1.66)
0.5–11.5 (women)/22.5 (men)	1.00	1.00	1.00	1.00
12(women)/23 (men) or more	1.53 (1.18–1.99)	1.57 (1.21–2.04)	1.56 (1.20–2.02)	1.46 (1.12–1.90)
*Binge drinking*				
Non-drinker	1.43 (1.03–1.97)	1.42 (1.02–1.96)	1.42 (1.03–1.96)	1.39 (1.00-1.92)
No bingeing	1.00	1.00	1.00	1.00
Less often than once a week	1.27 (1.06–1.52)	1.26 (1.06–1.51)	1.27 (1.06–1.52)	1.18 (0.98–1.41)
Once a week or more often	1.35 (1.02–1.77)	1.34 (1.02–1.77)	1.33 (1.01–1.76)	1.16 (0.87–1.54)
*Problem drinking*				
No	1.00	1.00	1.00	1.00
Yes	1.84 (1.54–2.19)	1.85 (1.55–2.20)	1.84 (1.54–2.19)	1.75 (1.46–2.09)

### Drinking patterns and antidepressant medication

Heavy drinking was associated with antidepressant medication (HR 1.53, 95% CI 1.18–1.99) compared to moderate drinking. (Table 3) Binge drinking showed an association with antidepressant medication compared to non-bingeing drinkers with a HR 1.27, CI 1.06–1.52 for individuals bingeing less often than once a week and a HR 1.35, CI 1.02–1.77 for individuals bingeing once per week or more often. Non-drinking was associated with antidepressant medication (HR 1.43, CI 1.03–1.97) compared to non-bingeing drinkers. Problem drinking showed an association with antidepressant medication (HR 1.84, CI 1.54–2.19). Adjustments for occupational position and marital status had negligible effects whereas adjustments for relative weight and health behaviours and for prior antidepressant medication slightly attenuated the associations concerning heavy amount of drinking and problem drinking and abolished the associations concerning binge drinking. The individual contributions of relative weight and health behaviours were also checked and smoking had the strongest contribution (data not shown). The associations between drinking patterns and antidepressant medication were also run using logistic regression and the results were largely similar.

Gender-stratified analyses between drinking patterns and antidepressant medication among participants without prior antidepressant medication are presented in Supplemental table S1. Heavy (1.63, 1.22–2.17), binge (1.43, 1.02–2.00) and problem (1.91 (1.56–2.33) drinking were all associated with antidepressant medication among women. Among men an association between problem drinking and antidepressant medication was observed (1.64, 1.15–2.36).

The vast majority of participants with prior antidepressant medication were moderate drinkers. Of them 14% were binge drinking once a week or more often and 28% were problem drinkers. There were no statistically significant associations between drinking patterns and subsequent antidepressant medication. (Table 4)

**Table 4:** The distributions of drinking patterns (n, %) among participants with prior antidepressant medication. The prevalence of antidepressant medication and the associations between drinking patterns and subsequent antidepressant medication. Odds ratios and their 95% confidence intervals

	*n*	%	% with antidepressant medication	Age + gender adjusted
*Weekly amount of drinking*				
0	60	8	68	1.10 (0.62–1.95)
0.5–11.5 (women)/22.5 (men)	598	83	66	1.00
12 or more (women)/23 or more (men)	66	8	74	1.47 (0.82–2.62)
p			0.701	
*Binge drinking*				
Non-drinker	62	9	69	1.36 (0.74–2.50)
No bingeing	253	35	62	1.00
Less often than once a week	301	42	68	1.28 (0.89–1.85)
Once a week or more often	100	14	73	1.69 (0.98–2.92)
p			0.095	
*Problem drinking*				
No	503	72	65	1.00
Yes	195	28	72	1.38 (0.96-2.00)
p			0.121	

## Discussion

This study sought to examine associations between drinking patterns and subsequent antidepressant medication among employees. Heavy, binge and problem drinking were all associated with antidepressant medication. The associations mainly remained after adjusting for age and gender, occupational position, marital status and for relative weight and health behaviors.

### Results in relation to previous studies

Heavy amount of drinking, binge drinking and problem drinking were all associated with antidepressant medication and the results thus support previous evidence showing that alcohol drinking is associated with depression [6–8]. Previous findings regarding various drinking patterns have been less clear supporting the associations between problem drinking and depression but varying concerning alcohol intake. A recent meta-analysis concluded that heavy drinking did not significantly predict occurrence of depressive symptoms after adjusting for potential confounders [8]. Many longitudinal studies among general populations have failed to find associations between heavy drinking and subsequent depression. Different cut-points for heavy drinking might partly explain the difference. For example, compared to our study two Spanish studies reporting no association used a relatively low cut-point for heavy drinking namely 15 g or over per day [15, 16]. A British study examined total weekly volume of alcohol consumed and drinking frequency but found no associations [11]. The cut-point for heavy drinking (exceeding 168 g per week for men and 112 g per week for women) was lower than that of our study. A study examining Chinese, English and U.S. cohorts reporting no association similarly had lower cut-points for heavy drinking (196 g per week for men and 98 g per week for women) [33] whereas another Finnish study reporting an association used nearly similar cut-points compared to us [10].

Some previous studies on general populations have suggested that binge drinking instead of heavy amount of drinking is associated with depression [18]. The results of the present study did not highlight the importance of binge or problem drinking since all studied drinking patterns showed an association. It might be that the measure of binge drinking with a cut-point of six units of alcohol on occasion did not sufficiently differentiate participants with severest binge drinking patterns. Another Finnish study examining the associations between binge drinking and depression measured alcohol intoxications, hangovers and pass-outs per year [10].

A major difference to previous studies was our register-based outcome. Previous studies on general populations have mainly used self-report measured of depression, which are likely to overestimate the prevalence of depression [2]. Antidepressant medication is likely to cover at least severe cases of depression as current guidelines recommend medical therapy in such cases [3]. Our results are contrasting to a previous Danish study, which examined antidepressant medication prescription and found an association between alcohol drinking and antidepressant medication prescription only when age and gender were adjusted and when controlling additionally for smoking status, cigarettes per day, physical activity, education, income, civil status, body mass index and plasma C-reactive protein no association was found [14]. In contrast to us the study only examined weekly alcohol intake and did not include information on problem and binge drinking.

In line with previous studies [11, 14–16, 33–35] non-drinking was associated with antidepressant medication. Instead of moderate alcohol drinking protecting from depression the association likely result from other characteristics of non-drinkers. The group of non-drinkers is likely to include individuals with a history of problem drinking and individuals with illnesses [36, 37]. In the British study the association between non-drinking and depression was abolished after controlling for baseline self-reported health [11]. The Norwegian study found that excluding previous heavy drinkers accounted for about one-third of the association [34] and a U.S study found no association between lifetime non-drinking and depression [38]. In our study non-drinking was measured by non-drinking during the baseline survey and we had no information about the previous drinking history.

The association between alcohol drinking and depression is likely bidirectional, i.e. alcohol problems contribute to depression and depression contributes to alcohol problems [17]. To examine the association of drinking patterns contributing to antidepressant medication we excluded participants with a prior antidepressant medication and our results thus supported the direction of association from alcohol drinking to depression. Additional analyses among participants with recent antidepressant medication did not show statistically significant associations probably at least partly due to limited statistical power. A recent U.S study found that risk of developing depression increased gradually with the severity of alcohol use disorder at baseline and thus supported the direction of the association from alcohol drinking to depression [38]. All in all, the results encourage to inquire about alcohol drinking when meeting patients with symptoms of depression.

Previous studies have suggested associations between alcohol drinking and depression to be stronger among women than men [8, 17]. In our study no significant interactions were observed and women and men were combined. The cut-points for heavy drinking and problem drinking were, however, lower for women. Lower cut-points were chosen since there were considerable differences in the drinking patterns between women and men and the Finnish guidelines suggested different cut-points.

To shed light on potential mechanisms behind the associations the models were adjusted for factors associated both with alcohol drinking and depression namely occupational position, marital status and relative weight, smoking and physical activity. Relative weight and health behaviours minimally attenuated the associations concerning heavy and problem drinking and abolished the associations concerning binge drinking. Of relative weight and health behavior measures smoking had the strongest contribution. Otherwise the adjustments did not contribute to the associations suggesting that these factors were unimportant in mediating between the association between alcohol drinking and antidepressant medication.

### Methodological considerations

The strengths of the study include a large data set, a prospective design, several variables on alcohol drinking and a register-based measure of depression. However, part of depression cases treated without medication might not be included and the outcome might instead include cases of anxiety disorders, sleep problems and chronic pain as antidepressant medication is used for those purposes as well. However, overall antidepressants are mainly used for treating depression [5, 39]. A further strength was the possibility to exclude participants with prior antidepressant medication although some participants may have had depression even earlier as depression is recurrent by nature. Of the original sample of 8960 employees 6459 were included in the study. A major reason for exclusion was not consenting to register data linkage. According to non-response analysis men in lower occupational positions and employees with medically-certified sickness absence were somewhat overrepresented among the non-consenters but the differences were small and not fully consistent [26]. All the participants were employed at baseline and thus individuals with severe mental or other health problems leading to work disability have likely selected out and our results might thus underestimate the real situation.

## Conclusions

Our study showed that alcohol drinking is associated with antidepressant medication among employees. The association is not limited to problem drinking. Reducing heavy drinking, binge drinking and problem drinking might help prevent depression.

## Supplementary Information

Below is the link to the electronic supplementary material.

Supplementary Material 1

## Data Availability

The Helsinki Health Study data cannot be made publicly available due to strict data protection laws and regulations. The data can only be used for scientific research. More information on the survey data can be requested from the Helsinki Health Study research group (kttl-hhs@helsinki.fi).
