# Supplementary material for: Drinking patterns and antidepressant medication – a prospective register-linked study among 40 to 60-year-old employees
Source: Soc Psychiatry Psychiatr Epidemiol. 2025 Sep 27;61(10):2147–54. doi: 10.1007/s00127-025-02996-z (PMC13586030; doi:10.1007/s00127-025-02996-z)
Supplement: Supplementary file 1 [file 127_2025_2996_MOESM1_ESM.docx]

Supplemental Table S1. The associations between drinking patterns and subsequent antidepressant medication among women and men. Hazard ratios and their 95% confidence intervals.

|  | Model 1 = Age + gender | Model 1 + occupational position | Model 1 + marital status | Model 1 + bmi, smoking and physical activity |
| --- | --- | --- | --- | --- |
| **Women** |  |  |  |  |
| **Weekly amount of drinking** |  |  |  |  |
| 0 | 1.16 (0.83-1.62) | 1.15 (0.83-1.61) | 1.16 (0.83-1.62) | 1.18 (0.85-1.65) |
| 0.5-11.5 | 1.00 | 1.00 | 1.00 | 1.00 |
| 12 or more | 1.63 (1.22-2.17) | 1.67 (1.25-2.23) | 1.67 (1.25-2.22) | 1.56 (1.17-2.08) |
| **Binge drinking** |  |  |  |  |
| Non-drinker | 1.28 (0.89-1.85) | 1.27 (0.88-1.83) | 1.28 (0.88-1.85) | 1.26 (0.87-1.82) |
| No bingeing | 1.00 | 1.00 | 1.00 | 1.00 |
| Less often than once a week | 1.28 (1.06-1.54) | 1.27 (1.05-1.53) | 1.27 (1.05-1.53) | 1.20 (0.99-1.46) |
| Once a week or more often | 1.43 (1.02-2.00) | 1.43 (1.02-1.99) | 1.43 (1.02-2.00) | 1.26 (0.89-1.77) |
| **Problem drinking** |  |  |  |  |
| No | 1.00 | 1.00 | 1.00 | 1.00 |
| Yes | 1.91 (1.56-2.33) | 1.92 (1.57-2.34) | 1.91 (1.57-2.34) | 1.83 (1.50-2.25) |
| **Men** |  |  |  |  |
| **Weekly amount of drinking** |  |  |  |  |
| 0 | 1.45 (0.74-2.87) | 1.47 (0.74-2.91) | 1.45 (0.73-2.86) | 1.49 (0.75-2.95) |
| 0.5-22.5 | 1.00 | 1.00 | 1.00 | 1.00 |
| 23 or more | 1.18 (0.63-2.19) | 1.19 (0.64-2.21) | 1.17 (0.63-2.17) | 1.19 (0.64-2.21) |
| **Binge drinking** |  |  |  |  |
| Non-drinker | 2.37 (1.06-5.30) | 2.39 (1.06-5.36) | 2.39 (1.07-5.34) | 2.12 (0.94-4.78) |
| No bingeing | 1.00 | 1.00 | 1.00 | 1.00 |
| Less often than once a week | 1.40 (0.77-2.53) | 1.45 (0.80-2.63) | 1.46 (0.80-2.65) | 1.22 (0.67-2.22) |
| Once a week or more often | 1.39 (0.73-2.64) | 1.38 (0.72-2.61) | 1.41 (0.74-2.69) | 1.11 (0.57-2.14) |
| **Problem drinking** |  |  |  |  |
| No | 1.00 | 1.00 | 1.00 | 1.00 |
| Yes | 1.64 (1.15-2.36) | 1.64 (1.14-2.35) | 1.64 (1.14-2.35) | 1.50 (1.04-2.15) |
| **P-value for interaction between gender and drinking pattern** | | |  |  |
| **Weekly amount of drinking** | 0.585 |  |  |  |
| **Binge drinking** | 0.503 |  |  |  |
| **Problem drinking** | 0.367 |  |  |  |
